# Day-Time Patterns of Carbohydrate Intake in Adults by Non-Parametric Multi-Level Latent Class Analysis—Results from the UK National Diet and Nutrition Survey (2008/09–2015/16)

**DOI:** 10.3390/nu11102476

**Published:** 2019-10-15

**Authors:** Chaochen Wang, Suzana Almoosawi, Luigi Palla

**Affiliations:** 1 Department of Public Health, Aichi Medical University, Nagakute, Aichi 480-1195, Japan; chaochen@wangcc.me; 2 Department of Medical Statistics, London School of Hygiene & Tropical Medicine, London WC1E 7HT, UK; 3 NNEdPro Global Centre for Nutrition and Health, Cambridge CB4 0WS, UK; suzana.almoosawi@gmail.com

**Keywords:** diurnal patterns of eating, NDNS RP, multilevel latent class analysis, chrononutrition, nutritional epidemiology

## Abstract

This study aims at combining time and quantity of carbohydrate (CH) intake in the definition of eating patterns in UK adults and investigating the association of the derived patterns with type 2 diabetes (T2D). The National Diet and Nutrition Survey (NDNS) Rolling Program included 6155 adults in the UK. Time of the day was categorized into 7 pre-defined time slots: 6–9 am, 9–12 noon, 12–2 pm, 2–5 pm, 5–8 pm, 8–10 pm, and 10 pm–6 am. Responses for CH intake were categorized into: no energy intake, CH <50% or ≥50% of total energy. Non-parametric multilevel latent class analysis (MLCA) was applied to identify eating patterns of CH consumption across day-time, as a novel method accounting for the repeated measurements of intake over 3–4 days nested within individuals. Survey-designed multivariable regression was used to assess the associations of CH eating patterns with T2D. Three CH eating day patterns (low, high CH percentage and regular meal CH intake day) emerged from 24,483 observation days; based on which three classes of CH eaters were identified and characterized as: low (28.1%), moderate (28.8%) and high (43.1%) CH eaters. On average, low-CH eaters consumed the highest amount of total energy intake (7985.8 kJ) and had higher percentages of energy contributed by fat and alcohol, especially after 8 pm. Moderate-CH eaters consumed the lowest amount of total energy (7341.8 kJ) while they tended to have their meals later in the day. High-CH eaters consumed most of their carbohydrates and energy earlier in the day and within the time slots of 6–9 am, 12–2 p.m. and 5–8 pm, which correspond to traditional mealtimes. The high-CH eaters profile had the highest daily intake of CH and fiber and the lowest intake of protein and fat. Low-CH eaters had greater odds than high-CH eaters of having T2D in self-reported but not in previously undiagnosed diabetics. Further research using prospective longitudinal studies is warranted to ascertain the direction of causality in the association of CH patterns with type 2 diabetes.

## 1. Introduction

Timing of eating is believed to affect a wide variety of physiological processes, especially glucose metabolism [1]. It is also a potential modifiable behavioral factor that may explain why shift workers have a higher risk of developing metabolic syndrome [2] and type 2 diabetes (T2D) [3]. However, to date inconsistencies remain in how diurnal eating patterns (DEP) could be derived and their association with health outcomes analyzed. Accordingly, a recent analysis of the Australian National Nutrition and Physical Activity Survey utilized latent class analysis to derive discernible DEPs [4]. Such data-driven approach represents a more comprehensive way of describing DEPs and their potential association with health. Yet, day-to-day variation in DEPs and timing of nutrient intake were not accounted for and warrant further investigation given that diurnal variations in glucose and lipid metabolism have been established [5].

Food is increasingly recognized as a natural zeitgeber with carbohydrate (CH), in particular, being known to entrain the clock system through its effect on glucose metabolism and insulin secretion [6,7]. Timing of CH intake has been shown to influence multiple physiological processes and health outcomes. Replacing fat or protein with CH in the morning has been found to be inversely associated with metabolic syndrome in a longitudinal analysis of the 1946 British Birth Cohort [8]. While in a randomized controlled trial eating high-glycemic index CH late in the day induced a higher postprandial glycemic control compared to when it was consumed in the morning [9]. A more recent study indicated that lowering CH intake at breakfast while increasing fat intake improves glycemic variability over the day [10]. Further studies have indicated that consuming CH at night at 23:00 worsens postprandial glucose profile the following morning compared to when the same amount is consumed earlier in the evening at 18:00 [11]. Whether the amount, the timing of CH consumption during the day or both are critical to health is an important question to address for guiding recommendations on optimal dietary intake.

Hence, in the present article, we aimed first at characterizing both time and quantity patterns of CH consumption in a nationally representative sample of British adults from the National Diet and Nutrition Survey Rolling Program (2008–2016), taking into account the variability of nutrient intake across days of dietary records via a multilevel latent class analysis method that has never been applied to nutrition surveys. Second, we aimed at testing the association between the derived pattern with diabetes (self-reported and/or based on fasting glucose and hemoglobin A1c, HbA1c). 

## 2. Materials and Methods

### 2.1. Study Population

The UK National Diet and Nutrition Survey Rolling Program (NDNS RP 2008/09–15/16) [12] is designed as a nationally representative sample to assess the diet, nutrient intake and nutritional status of the general population living in the UK. Details of the rationale, design and methods of the survey have been described elsewhere [13,14]. In brief, for years 1 to 8 combined, a sample of 39300 addresses was randomly selected from 1438 postcode sectors or Primary Sampling Units (PSUs). Response rates for completion of the required diet diary (three to four consecutive days) were 56%, 53% and 53% for years 1 to 4, 5 to 6 and 7 to 8, respectively. A total of 6155 adults (2537 men and 3618 women, age ≥ 19-year-old) were retained in the current study. Ethical approval for the NDNS RP was obtained from the Oxfordshire Research Ethics Committee [13].

### 2.2. Diet Diary and Definition of CH Intake

Participants were asked to keep a record of everything eaten or drunk over four consecutive days. Accordingly, there were 24,483 observation days collected in the NDNS RP dataset. In the diet diary, participants were also asked to write down the portion sizes in household measures. Detailed diary checking was performed by trained staff to code and convert the food consumption into energy and nutrients intake using the DINO (Diet in Nutrients Out) [15] system. Intakes of nutrients were calculated from the food consumption records using a specially adapted Nutrient Databank [16] originally developed for the Dietary and Nutritional Survey of British Adults [17]. Further details of data coding and editing are outlined in Appendix A of the NDNS RP official reports [13,14].

The consumption of CH was defined as total sugar plus starch intake (gram), fiber was not included as it was deemed not to contribute any energy intake in the NDNS RP data [18]; time across a typical survey day was classified according to the standard NDNS classification which consists of 7 pre-defined time slots: 6–9 am, 9–12, 12–2 pm, 2–5 pm, 5–8 pm, 8–10 pm, 10 pm–6 am; CH consumption in each time-slot was further categorized as: no energy intake occurred; CH contributed <50% or ≥50% of energy intake within that time slot, where these thresholds reflect the current UK recommendation for daily CH intake [19].

### 2.3. Statistical Analysis

Latent class analysis (LCA) is a statistical technique that performs unsupervised classifications based on observed categorical variables [20]. Multilevel latent class analysis (MLCA) [21,22,23] is an extension of LCA which allows for the repeated measurement of categorical variables, here represented by CH intake over 3–4 days nested within individuals. MLCA extracts day-level latent classes which are then used as indicators for a second latent class model at the individual level. Therefore, under MLCA models, individuals are allowed to have different probabilities of following alternative DEPs during their survey period. In particular, we employed non-parametric MLCA approach which relaxed the assumption that the multi-level model random intercepts follow a normal distribution and is less computationally demanding [21,24].

The following sequential modelling strategy was applied [22]: First, a series of traditional LCA models were fitted to determine the number of classes at the day-level, ignoring the hierarchical structure of the observations; next, a series of MLCA models were fitted to derive the individual-level classes accounting for such hierarchical structure; last, sensitivity analyses were conducted to investigate the effect of changing day level classification on the individual level classification. For both LCA and MLCA, the class assignment was determined by the maximum posterior probability given the model using the Bayes theorem.

The number of classes at day level was selected by the following rules: 1) The evaluation of model fit indices, including the Bayesian information criterion (BIC) and entropy; 2) the Lo-Mendell-Rubin Likelihood Ratio Test (LMR-LRT) [25,26] which compares *q* vs. *q*-1 classes models, where *q* is the number of latent classes; and most importantly; 3) clear class separation as well as pattern interpretability. LCA and MLCA models were fitted in MPlus (Version 8,3, Muthén & Muthén: Los Angeles, CA, USA) [27]. Example Mplus codes for fitting the MLCA model are given in Appendix A.

### 2.4. Characteristics and Associations between CH Intake Classes and T2D

Background information of the participants on socio-demographic variables were collected (including smoking habits, ethnicity, education level, living with a partner or not, etc.). Participants also had their height, weight and waist circumferences (WC) measured during the interview visit. Recent Physical Activity Questionnaire (RPAQ) [28] was used to estimate physical activity. Blood samples were taken from the participants to measure their fasting blood glucose and HbA1c. T2D cases were defined if any of the following criteria were met: 1) fasting blood glucose ≥7 mmol/L; 2) HbA1c ≥6.5%; 3) self-report of being diagnosed with T2D. We also identified undiagnosed T2D participants if they met either of the first two criteria but did not report having T2D.

Individual-level point estimates of characteristics and 95% confidence intervals (CIs) were estimated by applying the relevant sampling weights [15,16]. Descriptive statistics of participants were presented as weighted means (95% CI) or weighted percentages (95% CI). Fasting blood glucose, HbA1c and average physical activity duration (hours-per-day) were log-transformed to improve normality. Weighted estimates of energy consumption (and their composition) across the 7 day-time slots were calculated for each individual-level latent class.

Associations between individual-level CH classes and T2D were explored using survey-adjusted multivariable logistic regression models. Potential confounders were selected as covariates if they had an association with both individual-level CH classes and T2D. Consequently, a crude model; a model adjusted for age (continuous), sex (men, women), BMI (continuous), smoking status (past, current, never), total energy intake (continuous), alcohol intake (continuous); and a model adjusted for the same covariates except with BMI being replaced with WC were fitted. We also conducted the same analyses taking (i) only the self-reported T2D status as the outcome among the total sample (independently of their fasting glucose and HbA1c), or (ii) undiagnosed T2D (those diagnosed by either HbA1c or fasting glucose but did not report diabetes) to see whether diagnosis was associated with reported food choices in survey members. Except for MLCA, all the statistical analyses were performed in Stata (Version 15.1, StataCorp LLC, College Station, TX, USA) [29].

## 3. Results

### 3.1. CH Eating Patterns in Day and Individual Levels

**Figure 1:**
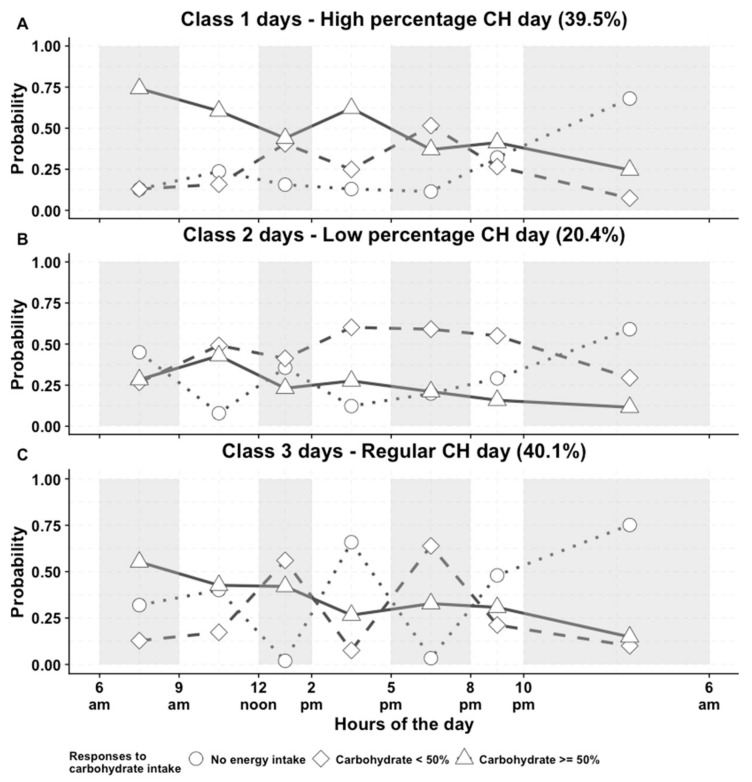
Day level latent classes solution for carbohydrate (CH) diurnal eating patterns: (**A**), high percentage CH day; (**B**), low percentage CH day; (**C)**, regular meal CH day. Grey, and white shades indicate the 7 time slots; Carbohydrate (CH) <50% indicates CH contributed less than 50% total energy intake; Carbohydrate ≥50% indicates CH contributed higher or equal to 50% total energy intake.

The majority of the participants (*n* = 6031, 97.9%) succeeded to provide a 4-day diary for the current analyses. More women (*n* = 78, 2.2%) than men (*n* = 46, 1.8%) discontinued their diary on day 3 or skipped 1 day of their diet diary. After examining and comparing details of different solutions (discussion of how the results were chosen, and figures from other competing solutions are presented in Appendix B), three CH eating day patterns emerged from the collected diet diaries (Figure 1).

Class 1 (Figure 1A) was defined as “high percentage CH day” since the probabilities (*p*) of CH contributing ≥50% of energy was relatively high across day time slots. In class 1 days, the probabilities were greater than 0.6 of having more than 50% of calories from CH between 6 a.m. to 9 a.m. (breakfast), 9 a.m. to 12 noon (morning snack) and 2 p.m. to 5 p.m. (afternoon snack). Moreover, during late evening and night time, the probabilities of having CH rich food were still relatively high (0.412 and 0.246, respectively).

Class 2 days (Figure 1B) were named as “low percentage CH day” because between 9 a.m. and 10 pm, the probability of carbohydrates contributing <50% of energy was always the highest. In class 2 days, participants also tended to have morning snacks (*p* = 0.079 of no energy intake), although these may also be interpreted as having a long or late breakfast in these mornings. Also, the probability of no intake at night was the lowest for Class 2 days (*p* = 0.59).

Class 3 days (Figure 1C) were defined as “regular meal CH day” because: 1) The probability of no energy intake was virtually null at lunch (*p* = 0.019) and dinner time (*p* = 0.034); 2) the probabilities of no energy intake between 9 a.m. and 12 noon and between 2 p.m. and 5 p.m. were also the highest (*p* = 0.401 and *p* = 0.659, respectively).

Detailed characteristics of the three CH eating day patterns can be found in Supplementary Tables S1 and S2. In brief, consumption of total energy, CH, sugar, starch and non-milk extrinsic sugar was the highest among Class 1 days. On the other hand, intake of protein, fat, and alcohol were the highest in the Class 3 days. Therefore, we anticipated that if one’s diet diary contains higher proportions of Class 1 days, or lower proportions of Class 3 days he/she would probably consume more CH in general.

**Figure 2:**
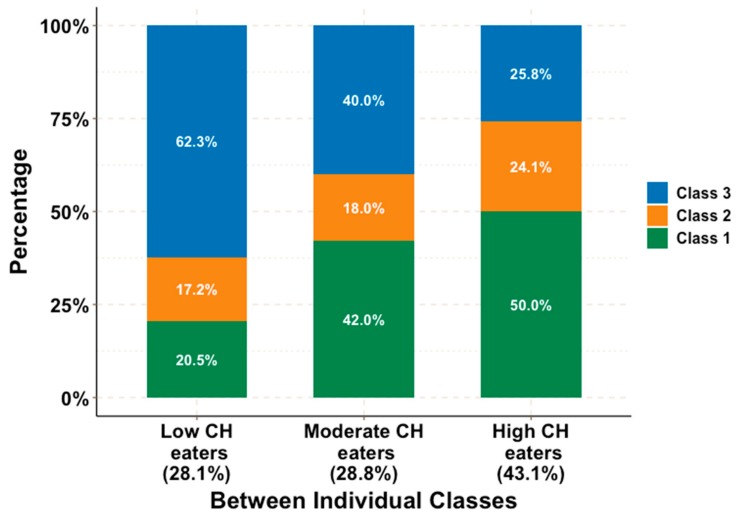
Multi-level latent classes solution for carbohydrate diurnal eating patterns: 3 classes in day level (Class 1 = high percentage CH days; Class 2 day = low percentage CH days; Class 3 = regular meal CH days), 3 classes in individual level.

Figure 2 showed the results from the chosen MLCA model where low-CH eaters (LCE, 28.1%), moderate-CH eaters (MCE, 28.8%) and high-CH eaters class (HCE, 43.1%) were defined. In the LCE group, 62.3% of days were classified as Class 3, and only 20.5% as Class 1. MCE had comparable proportions of day Class 1 (42%) and 3 (40%) and much lower one of Class 2 (18.0%). Among HCE, nearly 50% of their days were classified as Class 1 and approximately 25.8% of days of their dietary diary were of Class 3 and 24.1% were of Class 2. Individual level classification reflects differences in the probability of following a specific day-level class so that individuals with similar probabilities of following the day level classes were grouped together. During their 4-day-diary, all types of CH eaters have more than one class of CH eating day, and this is reflected in sizeable conditional probabilities (proportions) for each type of day.

Estimation of the population average nutrient intake is listed in Table 1. Specifically, the detailed consumption of CH over the time of day in the adult population and according to different types of CH eaters is estimated accounting for complex survey design. Overall, the targeted population in the survey consumed 7668.9 kJ on average, the contribution of their total energy from protein, fat, CH, and alcohol were 16.6%, 33.1%, 45.8%, and 4.4%, respectively. Total energy contributed by CH was close to 50% among HCE but was only 40.6% among LCE. In terms of components of CH consumed at each time slot, HCE consumed more than twice as LCE and nearly 4 times as MCE amount of sugar (37.9 g, 95% CI: 36.8, 39.2) and non-milk extrinsic sugar (i.e., free sugar, 11.1 g 95%CI: 10.7, 11.6) between 6–9 a.m. MCE consumed more sugar and starch outside the traditional meal times: between 9–12 noon, 2–5 p.m., 8–10 p.m. and 10 p.m.–6 a.m. The mean total fiber consumption was the highest for HCE (14.7 g, 95% CI: 14.4, 14.9) compared to MCE (12.5 g, 95%CI: 12.1, 12.9) and LCE (13.7 g, 95% CI: 13.4, 14.0).

**Figure 3:**
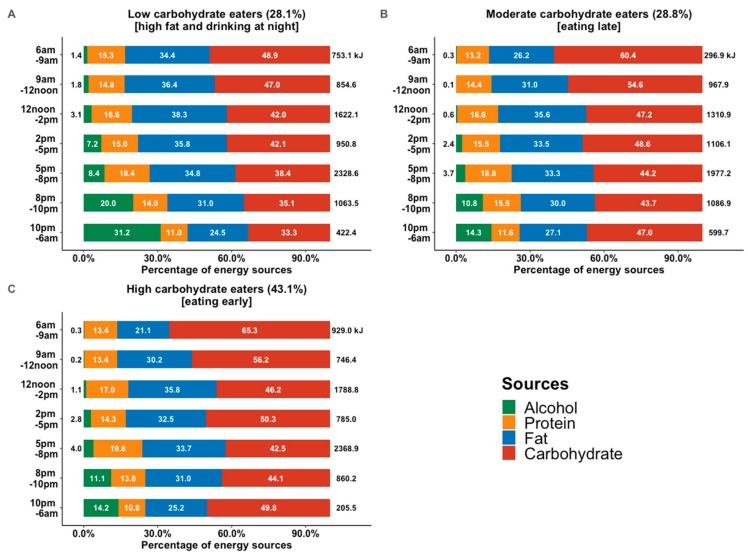
The compositions (%) and absolute (kJ) energy consumption within each time slot by individual level carbohydrate eating classes: (**A**), low carbohydrate eaters (LCE); (**B**), moderate carbohydrate eaters (MCE); (**C**), high carbohydrate eaters (HCE).

Weighted composition in total energy intake across the time of the day for three types of CH eaters are illustrated in Figure 3. LCE had the highest total energy intake (7985.8 kJ, 95%CI: 7283.3, 8146.3) while MCE had the lowest energy intake (7341.8 kJ, 95%CI: 7172.5, 7511.2, Table 1). Moreover, in LCE (Figure 3A), CH never contribute more than 50% of their total energy over the day and mostly had the highest energy from fat out of the 3 classes of eaters. Energy contributed by alcohol in LCE was the highest across time slots and considerably higher after 8pm, namely 20.0% (8–10 pm) and 31.2% (10–6 am).

On the other hand, the energy consumption in the MCE (Figure 3B) seemed to be shifted towards later in the day compared with HCE (Figure 3C). MCE tended to have later meals and they also had the highest total energy consumption (599.7 kJ) at night (10 pm–6 am) across all three types of eaters. HCE consumed the highest total energy (929.0 kJ) between 6 a.m. to 9 a.m. in the morning and the lowest total energy between 10 p.m. to 6 a.m. (205.5 kJ). Overall, HCE consumed their energy mainly at the time slots: 6–9 am, 12–2 p.m. and 5–8 pm.

### 3.2. Characteristics of the three types of CH eaters

Socio-demographic characteristics of UK adults according to their individual level latent class memberships are shown in Table 2. MCEs were relatively younger. Gender distribution across the three types of CH eaters was reasonably even. The distribution of the CH eaters appears to be changing over the year of survey (*p* = 0.015). LCE represented 32.5% of the population in 2008 but dropped below 30% in 2016. The proportion of HCE increased from 41.2% to the highest (50.6%) in 2009, then gradually declined to 38.4% in 2016.

On average, LCE had greater BMI (27.8 kg/m^2^) and WC (98.9/89.9 cm in men/women) compared with 27.2, 27.3 kg/m^2^, and 95.9/88.7, 98.1/87.2 cm respectively in MCE and HCE. MCE had the highest prevalence of being a current smoker (27.8%), shortest time of daily physical activity (geometric mean: 0.62, 95% CI: 0.57, 0.68, hours/day).

### 3.3. Association between the CH Eating Patterns and T2D

Associations of individual CH eating patterns with T2D are presented in Table 3. The prevalence of self-reported T2D was almost twice among LCE (10.6, 95%CI: 8.1, 13.8) compared to participants in HCE group. The adjusted odds of being self-reported T2D was 139% higher in LCE (OR = 2.39, 95% CI: 1.51, 3.77, *p* < 0.001), and 42% higher in MCE (OR = 1.42, 0.85, 2.37, *p* = 0.181) than in HCE. If the status of T2D considered blood tests additionally, 93 more T2D cases were identified (22, 22, and 49 undiagnosed T2D cases in LCE, MCE, and HCE, respectively). The adjusted odds of being T2D were attenuated but still 67% higher in LCE (OR = 1.67, 95% CI: 1.10, 2.52, *p* = 0.016), 22% higher in MCE (OR = 1.22, 95% CI: 0.77, 1.93, *p* = 0.407) than in HCE group. However, when the diagnosed T2D were excluded, the adjusted odds for undiagnosed T2D became 31% lower in LCE (OR = 0.69, 95% CI: 0.35, 1.35), and 15% lower in MCE (OR = 0.85, 95% CI: 0.39, 1.86) compared with HCE group.

## 4. Discussion

Using multilevel non-parametric LCA (MLCA) as a novel classification technique and data from the NDNS RP (2008–2016), we examined DEPs for CH firstly at the diet-diary day level. Then individual level CH eating classes were derived, providing an estimate of the probability that an individual follows the specific day patterns identified in the first step.

Three interpretable, distinct latent classes were found: high CH percentage; low CH percentage; and regular meal CH days. In addition, three types of eaters were defined depending on participant’s diet across 3–4 days: low, moderate, and high CH eaters. For the first time, as far as we know, the day-to-day dietary intake pattern variation within individuals was successfully captured taking into account the timing of eating. Results from the MLCA models showed that people were indeed changing their diet from day to day which also suggests that assuming a person will always follow a certain type of DEP is not appropriate.

LCE had the highest total energy intake over the day compared to other groups. Detailed profiling of energy composition revealed that LCE had a higher percentage of fat intake over the day and higher energy intake from alcohol after 8 pm. MCE had the highest energy intake after 10 p.m. and generally later than HCE. These MCE might correspond to the “late eaters” defined by previous studies [4,30].

Finally, the HCE had the highest total daily CH intake which was consumed mostly at traditional mealtimes. They had the least amount of energy after 8 p.m. which may correspond to “early eaters” found by previous studies [4,30]. Overall, high CH eaters appear to have a diet with the highest daily intake of CH and fiber and the lowest intake of protein, fat as well as alcohol compared with the other two eating patterns.

Low CH diets which restrict CH in favor of increased protein or fat intake are currently commonly prescribed for weight-loss [31]. However, meta-analyses of randomized trials show that short-term weight-loss effect occurs to a similar extent when following either a low CH or a balanced diet [32]. An updated prospective cohort study has found a U-shaped relationship between all-cause mortality and CH intake, in which life span is greatest among people with around 50% energy contributed by CH [33]. Another meta-analysis of observational studies also reported a significantly higher risk of all-cause mortality associated with low CH diets [34]. These findings suggested that the long-term effect of either low or high CH intake might not be beneficial. Our definition of low, moderate, and high CH eaters has extended this concept of CH eating pattern into a diurnal context, investigating the potential usefulness of evaluating the effect of CH intake from another dimension—the timing of consumption—which has not been addressed so far in the literature.

In relation to health outcomes, LCE had higher prevalence of diabetes compared to MCE and HCE. This association was strong for self-reported T2D even after adjustment for potential confounders. Differences in the opposite direction were observed once the analysis was restricted to undiagnosed diabetes (although the small sample of undiagnosed participants is likely to have rendered the relationship nonsignificant), suggesting the possibility of reverse causality in our findings. Once survey members became aware of their T2D status, they may have switched their diet to a relatively lower percentages of CH or replaced CH with other types of energy sources such as fat or even alcohol.

Whether the effect of diurnal variation in intake of energy, fat and alcohol may have also contributed to diabetes or other health outcomes will need to be addressed by longitudinal studies. In a recent review, high-fat diets have been described to reduce the amplitude of clock genes, associated with glucose metabolism, in animal models [5]. Likewise, in humans, high energy intake in the evening has been associated with obesity [5]. In the current study, MCE had the highest energy intake after 10 p.m., yet the prevalence of undiagnosed diabetes was lower compared to HCE. MCE, however, differed from LCE in the composition of their night-time eating occasions, highlighting the need to characterize evening and night meals that may induce adverse effects on health. Likewise, our analyses describing different diurnal CH eating patterns revealed the complexity of eating behaviors in the population and the utility of exploratory, data-driven methods in capturing DEP [35].

### Limitations and Strengths

Some statistical and epidemiological limitations in the current study merit consideration. First, we ignored the order of observation days in the MLCA models, treating the observation days as exchangeable repeated measures. However, other statistical techniques which could take the order or the longitudinal nature of the data into account, such as repeated measures latent class analysis (RMLCA) [22], latent transition analysis (LTA) [22], or latent class growth analysis (LCGA) [36,37,38] are not applicable for the NDNS RP dataset as they would require that the 3 or 4 repeated measurements of diet diaries be recorded at the same time points longitudinally.

Second, the classification of individuals to latent CH eating classes was defined by maximum posterior probability assignment rule whereby individuals are assigned to the class with the highest posterior probability of membership [39], ignoring the uncertainty in the classification. However, the maximum probability rule is still able to minimize the number of incorrect assignments [40,41], compared to the alternative approach of multiple pseudo-class draws [42]. A Monte Carlo simulation study [41] also found that an inclusive LCA (i.e., LCA with relevant covariates) would probably perform better than non-inclusive LCA and has the potential to reduce bias of class assignment. However, whether this advantage can be extended to MLCA in the current study is unknown.

Third, it is not possible to differentiate between under-reporting due to ill health vs. actual misreporting in data from NDNS RP. Consequently, most previous studies using data from the NDNS RP do not adjust for under-reporting of nutrients intake in the models. Detailed data on shift work and chronotype were also not available to explore whether diurnal eating patterns differed by shift work or chronotype. Moreover, the current study only focused on characterizing patterns of CH intake, as an example of a macronutrient that may potentially influence circadian rhythms. Yet, other macronutrients may equally play a role in entraining the clock system [7]. Our study also did not take into account differences in CH quality in deriving the CH diurnal patterns, although we did find that LCE were consuming less “good quality” CH than HCE. Experimental studies in animal models have previously reported that rapidly digestible starch has a stronger entraining effect on peripheral clocks than slowly digestible starch [43]. Consequently, future studies should address how timing of intake of different types of carbohydrates may influence both short-term physiological responses and long-term health outcomes.

Fourth, another important limitation is the cross-sectional study design which does not permit assessment of causal relationship between the CH eating patterns and the surveyed health outcomes. However, the sensitivity analyses we conducted on undiagnosed diabetics were indeed suggesting the presence of reverse causation (with behavior change as a result of health concerns) since HCE were in that case associated with greater prevalence of diabetes.

Finally, the findings here may not be generalizable to populations in other countries. However, datasets from other national and cultural context with a day temporal structure as well as repeated measurements in time could be explored by this approach and help us understand and summarize the complexity in human eating behavior.

Strengths of this study include the large, nationally representative sample of UK adults. The classification process through model-based, data-driven procedures minimizes reliance on researchers’ normative notions of healthy dietary patterns and can highlight multivariate/complex features that may otherwise be overlooked [44]. MLCA especially accounted for the multilevel structure of the data, where the 4-day diet diaries were nested within participants, capturing the day-to-day variation of the respective CH eating patterns within individuals.

## 5. Conclusions

We have successfully defined CH eating patterns in the general population in UK adults using the NDNS RP database at both day and individual observation level. MLCA proved to be a useful exploratory technique and extracted 3 subgroups from the 2008-16 nutrition survey which we broadly defined as low, moderate and high CH eaters. LCE tended to have higher energy intake from fat and alcohol compared to other types of CH eaters, especially during late night-time. Moderate CH eaters reported the lowest total daily energy intake and tended to shift their energy intake towards later in the day. High CH eaters obtained most of their CH and energy earlier in the day and ate mostly at traditional mealtimes. These CH patterns differed by timing, absolute and relative contributions to energy consumption. LCE had higher odds of being diagnosed with T2D than HCE based on self-reported diabetes, while the association became weaker when undiagnosed T2D were also included, pointing towards potential changes in dietary intake once individuals become aware of their T2D status. Longitudinal studies are warranted to investigate whether the identified diurnal eating patterns themselves are changing over time and to study how such patterns may relate to changes of blood pressure, obesity and other health outcomes over the life-course.

## Figures and Tables

**Table 1:** Weighted means and percentages (95% CIs) of the macronutrients intake according to individual level carbohydrate eating classes, and details of carbohydrate intake across the 7 time-slots over the day. (NDNS RP 2008/09–15/16).

	Total	LCE	MCE	HCE
*n*	6155	1730	1772	2653
Total energy (kJ)	7668.9 (7583.1, 7754.7)	7985.8 (7823.3, 8146.3)	7341.8 (7825.3, 8146.3)	7677.8 (7555.8, 7799.8)
Protein (g)	74.2 (73.3, 75.1)	79.9 (77.9, 81.8)	69.3 (67.6, 71.0)	73.7 (72.5, 74.8)
Protein-%	16.6 (16.5, 16.8)	17.2 (16.9, 17.5)	16.3 (16.0, 16.6)	16.5 (16.3, 16.6)
Fat (g)	67.7 (66.8, 68.6)	74.7 (73.1, 76.4)	63.8 (62.1, 65.5)	65.7 (64.4, 67.0)
Fat- %	33.1 (32.9, 33.4)	35.4 (34.9, 35.8)	32.5 (32.1, 32.9)	32.0 (31.7, 32.3)
Alcohol (g)	12.8 (11.9, 13.7)	20.8 (18.3, 23.2)	10.7 (9.4, 11.9)	8.9 (8.1, 9.8)
Alcohol- %	4.4 (4.1, 4.6)	6.8 (6.3, 7.4)	3.8 (3.4, 4.3)	3.2 (2.8, 3.4)
Carbohydrate (g)	220.7 (218.2, 223.2)	203.8 (199.8, 207.8)	218.3 (212.9, 223.7)	233.4 (229.6, 237.2)
Carbohydrate- %	45.8 (45.6, 46.0)	40.6 (40.2, 41.0)	47.3 (46.8, 47.8)	48.3 (47.9, 48.6)
Total fiber (g)	13.8 (13.6, 13.9)	13.7 (13.4, 14.0)	12.5 (12.1, 12.9)	14.7 (14.4, 14.9)
Total sugar (g)	95.2 (93.6, 96.7)	83.4 (81.1, 85.7)	92.2 (88.9, 95.6)	104.9 (102.5, 107.3)
Total NME sugar (g)	56.9 (55.5, 58.3)	46.1 (44.1, 48.0)	60.7 (57.5, 63.8)	61.5 (59.4, 63.6)
Total starch (g)	125.4 (123.9, 126.9)	120.3 (117.7, 122.9)	125.9 (122.8, 129.2)	128.4 (126.2, 130.6)
6 am–9 a.m.	26.1 (25.3. 26.9)	23.0 (21.8, 24.3)	11.2 (10.0, 12.3)	37.9 (36.8, 39.2)
Fiber (g)	1.4 (1.3, 1.5)	1.4 (1.3, 1.5)	0.6 (0.5, 0.7)	2.0 (1.9, 2.2)
Sugar (g)	12.9 (12.4, 13.3)	10.2 (9.6, 10.9)	5.3 (4.8, 5.8)	19.7 (19.0, 20.4)
NME sugar (g)	7.1 (6.8, 7.3)	4.7 (4.3, 5.1)	3.2 (2.9, 3.6)	11.1 (10.7, 11.6)
Starch (g)	13.2 (12.7, 13.6)	12.8 (12.0, 13.5)	5.9 (5.1, 6.6)	18.3 (17.6, 19.1)
9 am–12 noon	27.8 (27.1, 28.6)	25.1 (23.9, 26.3)	33.0 (31.4, 34.6)	26.2 (25.1, 27.2)
Fiber (g)	1.4 (1.3, 1.5)	1.5 (1.4, 1.6)	1.6 (1.5, 1.7)	1.3 (1.2, 1.3)
Sugar (g)	13.9 (13.5, 14.3)	11.6 (10.9, 12.3)	15.7 (14.8, 16.6)	14.2 (13.6, 14.8)
NME sugar (g)	7.8 (7.5, 8.1)	5.7 (5.2, 6.2)	9.6 (8.9, 10.2)	8.1 (7.7, 8.5)
Starch (g)	13.9 (13.5, 14.4)	13.5 (12.8, 14.3)	17.3 (16.4, 18.3)	11.9 (11.3, 12.6)
12 noon–2 p.m.	45.3 (44.4, 46.2)	42.6 (40.9, 44.3)	38.7 (37.0, 40.4)	51.6 (50.2, 52.9)
Fiber (g)	3.1 (3.0, 3.2)	3.1 (2.9, 3.2)	2.3 (2.2, 2.5)	3.6 (3.5, 3.7)
Sugar (g)	16.8 (16.3, 17.2)	14.7 (14.0, 15.4)	14.9 (14.0, 15.7)	19.4 (18.7, 20.0)
NME sugar (g)	9.1 (8.8, 9.4)	7.3 (6.7, 7.8)	9.1 (8.4, 9.8)	10.3 (9.8, 10.8)
Starch (g)	28.5 (27.9, 29.2)	27.9 (26.6, 29.1)	23,8 (22.6, 24.9)	32.2 (31.2, 33.1)
2 pm–5 p.m.	27.3 (26.5, 28.2)	25.0 (23.6, 26.4)	33.6 (31.6, 35.6)	24.7 (23.6, 25.7)
Fiber (g)	1.5 (1.4, 1.6)	1.6 (1.5, 1.7)	1.9 (1.7, 2.0)	1.3 (1.2, 1.4)
Sugar (g)	13.3 (12.9, 13.7)	11.9 (11.3, 12.7)	14.5 (13.5, 15.5)	13.4 (12.8, 13.9)
NME sugar (g)	8.5 (8.2, 8.9)	6.9 (6.4, 7.5)	9.9 (9.0, 8.6)	8.6 (8.2, 9.1)
Starch (g)	14.0 (13.3, 14.6)	13.1 (12.1, 13.9)	19.1 (17.7, 20.4)	11.3 (10.6, 11.9)
5 p.m.–8 p.m.	58.5 (57.4, 59.7)	55.9 (54.1, 57.9)	54.6 (52.1, 57.0)	62.9 (61.3, 64.4)
Fiber (g)	4.4 (4.3, 4.5)	4.4 (4.2, 4.5)	3.7 (3.5, 3.9)	4.9 (4.7, 5.0)
Sugar (g)	19.9 (19.5, 20.5)	18.7 (17.9, 19.5)	18.6 (17.6, 19.5)	21.8 (20.9, 22.5)
NME sugar (g)	11.4 (11.0, 11.9)	10.2 (9.6, 10.8)	11.8 (10.9, 12.6)	12.1 (11.4, 12.7)
Starch (g)	38.5 (37.7, 39.4)	37.3 (35.8, 38.8)	35.9 (34.1, 37.9)	41.1 (39.9, 42.2)
8 pm–10 p.m.	25.3 (24.4, 26.2)	23.3 (21.9, 24.6)	29.7 (27.6, 31.7)	23.7 (22.5, 24.9)
Fiber (g)	1.4 (1.3, 1.5)	1.4 (1.3, 1.6)	1.6 (1.5, 1.8)	1.3 (1.5, 1.8)
Sugar (g)	12.2 (11.8, 12.6)	10.9 (10.3, 11.5)	13.2 (12.2, 14.2)	12.4 (11.8, 13.0)
NME sugar (g)	8.4 (7.9, 8.7)	7.3 (6.8, 7.8)	9.4 (8.5, 10.4)	8.3 (7.8, 8.8)
Starch (g)	13.1 (12.5. 13.7)	12.3 (11.4, 13.3)	16.4 (15.0, 17.8)	11.3 (10.5, 12.1)
10 p.m.–6 a.m.	10.3 (9.5, 11.1)	8.8 (7.7, 9.8)	17.6 (15.2, 19.9)	6.4 (5.8, 7.1)
Fiber (g)	0.41 (0.37, 0.45)	0.34 (0.29, 0.39)	0.74 (0.63, 0.85)	0.24 (0.21, 0.27)
Sugar (g)	6.2 (5.7, 6.7)	5.3 (4.6, 6.1)	10.0 (8.6, 11.5)	4.1 (3.7, 4.5)
NME sugar (g)	4.6 (4.1, 5.0)	3.9 (3.3, 4.6)	7.7 (6.4, 8.9)	2.9 (2.6, 3.3)
Starch (g)	4.1 (3.7, 4.6)	3.5 (2.9, 3.9)	7.5 (6.3, 8.8)	2.3 (1.9, 2.7)

Abbreviations: CI, confidence interval; NDNS RP, National Diet and Nutrition Survey Rolling Program; LCE, low carbohydrate eaters; MCE, moderate carbohydrate eaters; HCE, high carbohydrate eaters; NME sugar, non-milk extrinsic sugar. Non-milk extrinsic sugar is defined as additional added free sugar, such as table sugar, honey, glucose, fructose and glucose syrups, sugars added to food and sugars in fruit juices.

**Table 2:** Weighted means, percentages (95% CIs) of the according to individual level carbohydrate eating classes in the UK adults. (NDNS RP 2008/09 2015/16, *n* = 6155).

	LCE	MCE	HCE	
	(*n* = 1730)	(*n* = 1772)	(*n* = 2653)	*p*-Value ^1^
Total ^2^ (%)	28.3 (26.9, 29.9)	28.7 (27.1, 30.3)	43.0 (41.3, 44.7)	
Age (years)	51.0 (49.9, 52.1)	40.3 (39.1, 41.6)	51.7 (50.7, 52.7)	<0.001
Sex (%)				0.119
Men	50.0 (46.9, 53.1)	50.2 (47.0, 53.5)	46.6 (44.0, 49.1)	
Women	50.0 (46.9, 53.1)	49.8 (46.5, 53.0)	53.4 (50.9, 56.0)	
Survey years (% in rows)				0.015
1	32.5 (28.4, 36.9)	26.3 (21.9, 31.2)	41.2 (36.6, 46.0)	
2	26.8 (22.6, 31.3)	22.6 (18.6, 27.3)	50.6 (45.8, 55.4)	
3	22.6 (18.8, 26.9)	33.7 (28.6, 39.2)	43.6 (38.7, 48.7)	
4	27.9 (24.1, 32.2)	27.6 (23.8, 31.8)	44.4 (40.2, 48.7)	
5	27.9 (24.2, 32.0)	28.7 (24.4, 33.5)	43.3 (38.2, 48.6)	
6	28.0 (24.0, 32.4)	31.5 (26.9, 36.6)	40.5 (35.8, 45.3)	
7	29.1 (25.2, 33.4)	29.0 (24.5, 34.0)	41.8 (37.1, 46.7)	
8	31.1 (27.3, 35.3)	30.5 (25.9, 35.5)	38.4 (34.1, 42.8)	
Paid employment ^3^, Yes (%)	40.3 (37.0, 43.6)	40.8 (37.1, 44.5)	39.8 (37.1, 42.6)	0.907
Live with partner ^4^, Yes (%)	56.9 (53.6, 60.1)	38.4 (35.2, 41.8)	61.3 (58.7, 63.7)	<0.001
Household Income, GBP/year	36,558.5 (34,800.2, 38316.8)	27,180.8 (25,597.9, 28763.7)	32,171.6 (31,024.9, 33318.2)	<0.001
Ethnicity (%)				<0.001
White	94.2 (92.4, 95.6)	79.5 (76.4, 82.3)	91.9 (90.1, 93.4)	
Non-White	5.8 (4.4, 7.6)	20.5 (17.7, 23.6)	8.1 (6.6, 9.9)	
Education (%)				0.019
Degree or higher	29.0 (26.1, 32.1)	23.3 (20.5, 26.3)	26.2 (24.1, 28.5)	
Lower than degree	71.0 (67.9, 73.9)	76.7 (73.7, 79.5)	73.8 (71.5, 75.9)	
BMI (kg/m^2^)	27.8 (27.4, 28.2)	27.2 (26.7, 27.7)	27.3 (26.9, 27.6)	0.006
WC (cm)				
Men	98.9 (97.4, 100.5)	95.9 (94.1, 97.8)	98.1 (96.9, 99.2)	0.056
Women	89.9 (88.7, 91.3)	88.7 (87.1, 90.3)	87.2 (86.1, 88.2)	0.004
Physical Activity ^5^ (hours/day)	0.73 (0.68, 0.79)	0.62 (0.57, 0.68)	0.73 (0.68, 0.77)	0.005
Smoking status (%)				<0.001
Current	20.4 (18.0, 23.0)	27.8 (25.0, 30.9)	17.1 (15.4, 19.0)	
Ex-smoker	29.3 (26.5, 32.2)	16.8 (14.6, 19.2)	26.1 (24.9, 28.3)	
Never	50.3 (47.2, 32.2)	55.4 (52.2, 58.6)	56.8 (54.3, 59.3)	

Abbreviations: CI, confidence interval; NDNS RP, National Diet and Nutrition Survey Rolling Program; LCE, low carbohydrate eaters; MCE, moderate carbohydrate eaters; HCE, high carbohydrate eaters; BMI body mass index; WC, waist circumference; HbA1c, hemoglobin A1c. ^1^ For continuous variables, the *F* test was used to determine differences between latent classes with Bonferroni correction to account for multiple testing across >2 classes. For categorical variables, differences between latent classes were assessed using the adjusted Pearson $\chi^{2}$ test for survey data. ^2^ Total here indicates estimates of the percentages for each type of CH eaters among the population of UK adults; ^3^ Paid employment was defined as being in paid employment during the last 4 weeks prior to the survey; ^4^ Live with partner was defined as either living with a married husband/wife or a legally recognized civil partnership; ^5^ Physical activity was calculated as mean time spent at moderate or vigorous physical activity including both work-related and recreational activities during the survey.

**Table 3:** Odds ratios (OR) and 95% CI for low/moderate carbohydrate eating patterns versus high on T2D (self-reported or diagnosed by blood tests) in UK adults. (NDNS RP 2008/09–2015/16, *n* = 6155).

Outcomes	LCE		MCE		HCE
	(*n* = 1730)	*p*-Value ^1^	(*n* = 1772)	*p*-Value ^1^	(*n* = 2653)
**T2D (Self-reported, Yes)-Adjusted by Individual Weightings**					
*n*	85		55		72
% ^2^	10.6 (8.1, 13.8)		7.2 (5.1, 10.0)		5.8 (4.3, 7.7)
Crude model	1.94 (1.28, 2.95)	0.002	1.26 (0.80, 2.00)	0.321	1
Adjusted Model ^3^	2.33 (1.48. 3.69)	< 0.001	1.40 (0.83, 2.34)	0.203	1
Adjusted Model ^4^	2.39 (1.51, 3.77)	< 0.001	1.42 (0.85, 2.37)	0.181	1
**T2D (HbA1c** ≥**6.5% or Glucose** ≥**7 mmol/L, or Self-Reported)-Adjusted by Blood Weightings**					
N	107		77		121
% ^2^	12.2 (9.5, 15.4)		9.0 (6.6, 12.0)		8.5 (6.7, 10.6)
Crude model	1.49 (1.04, 2.15)	0.030	1.06 (0.73, 1.60)	0.780	1
Adjusted Model ^3^	1.66 (1.09, 2.52)	0.017	1.23 (0.78, 1.95)	0.375	1
Adjusted Model ^4^	1.67 (1.10, 2.52)	0.016	1.22 (0.77, 1.93)	0.407	1
**Undiagnosed T2D (HbA1c** ≥**6.5% or Glucose** ≥**7 mmol/L but Self-Reported No Diabetes)-Adjusted by Blood Weightings. Self-Reported T2D Cases Excluded.**					
*n*	22		22		49
% ^2^	2.8 (1.8, 4.6)		3.1 (1.7, 5.6)		4.2 (2.9, 5.9)
Crude model	0.71 (0.38, 1.33)	0.280	0.75 (0.38, 1.33)	0.420	1
Adjusted Model ^3^	0.76 (0.39, 1.50)	0.433	0.95 (0.45, 1.99)	0.885	1
Adjusted Model ^4^	0.69 (0.35, 1.35)	0.280	0.85 (0.39, 1.86)	0.688	1

Abbreviations: OR, odds ratio; CI, confidence interval; BMI, body mass index; WC, waist circumference; NDNS RP, National Diet and Nutrition Survey Rolling Program; LCE, low carbohydrate eaters; MCE, moderate carbohydrate eaters; HCE, high carbohydrate eaters; T2D, type 2 diabetes. ^1^ *p* values were obtained from Wald tests in logistic regression models adjusted for the complex survey design. ^2^ Percentages (95% CI) are adjusted by applying the individual or blood sample weights accordingly to reflect the proportion among the targeting population. ^3^ Adjusted for age (continuous), sex (men, women), BMI (continuous), smoking status (past, current, never), total energy intake (continuous), alcohol intake (continuous). ^4^ Adjusted for age (continuous), sex (men, women), WC (continuous), smoking status (past, current, never), total energy intake (continuous), alcohol intake (continuous).

**Figure A1:**
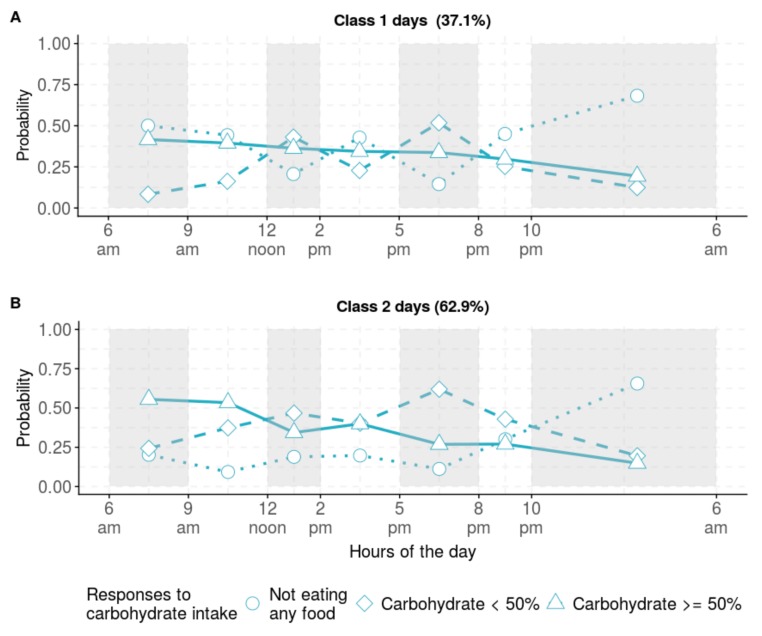
Day level latent classes solution for carbohydrate (CH) diurnal eating patterns: (**A**). Class 1 day is essentially similar to the regular meals CH day (RMD) shown in Figure 1C; (**B**). Class 2 day is a mixture of high percentage CH and low percentage CH days shown in Figure 1A,B. Grey, and white shades indicate the 7 time slots; Carbohydrate <50% indicates that within the time slots, carbohydrate contributed less than 50% total energy intake; Carbohydrate ≥50% indicates that within the time slots, carbohydrate contributed higher or equal to 50% total energy intake.

**Figure A2:**
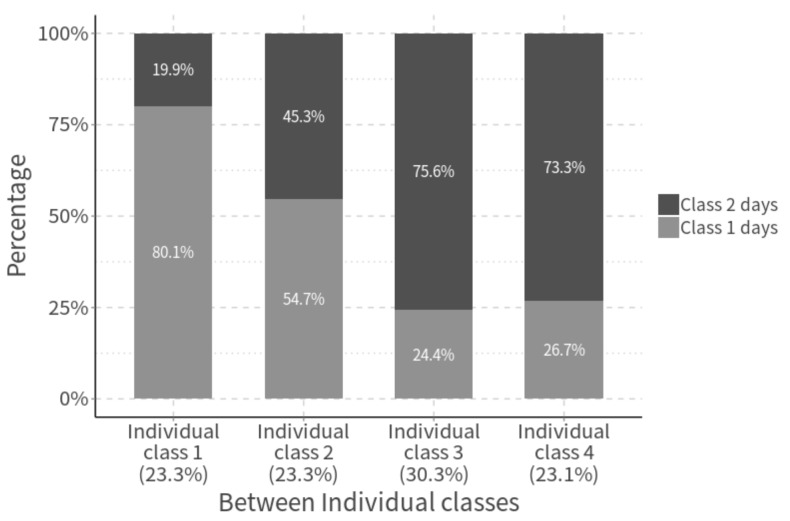
Multilevel Latent Class Model solution, 2 classes in day level, 4 classes in individual level. Individual class 3 and class 4 are essentially the same.

## Supplementary Materials

The following tables are available online at https://www.mdpi.com/2072-6643/11/10/2476/s1. Table S1: Day level latent class solution for three classes LCA model. (No individual level model, n = 24,483 observation days) Table S2: Means (standard deviations, sd), and counts (%) of the characteristics by different types of carbohydrate eating days (*n* = 24,483).

## Appendix A. Mplus Example Codes for Multilevel Latent Class Models

Mplus VERSION 8.3 (Linux) ! there are also Windows and Mac versions of the same program
MUTHEN & MUTHEN
05/06/2019 5:09 PM

INPUT INSTRUCTIONS

TITLE: 3-class at level 1 (CW), 3-classes at level 2 (CB) random effects model - non-parametric approach ordered polytomous variables for CH intake at each time slot over four days of NDNS survey 2008/09 - 2015/16
variable 0 = no energy intake
1 = eating & carb provided <50% calorie
2 = eating & carb provided ≥50% calorie

DATA: File is “../NDNS_Tslots.dat”; ! you will need to specify the path to your data
VARIABLE: NAMES = id id_dy Age Sex H6_9 H9_12 H12_14 H14_17 H17_20
H20_22 H22_6;
USEVAR = H6_9 H9_12 H12_14 H14_17 H17_20
H20_22 H22_6;
auxiliary = Age Sex;
CATEGORICAL = H6_9 H9_12 H12_14 H14_17 H17_20
H20_22 H22_6;
CLUSTER = id; !days nested within individuals
IDVARIABLE = id_dy;! Id with day number

BETWEEN = CB;

WITHIN = H6_9 H9_12 H12_14 H14_17 H17_20
H20_22 H22_6; ! time slot indicators for day level

CLASSES = CB(3) CW(3);

MISSING are .;

ANALYSIS:
type = twolevel mixture;
starts = 200 100; ! when increasing the n of classes, these numbers may need to be changed to larger ones
process = 8(starts); ! number of processors on your machine

MODEL:
%within%
%overall%

%between%
%overall%
CW ON CB;

Savedata:
file is “../NDNSslot_CW3CB3.txt”; ! you will need to specify the location to store the predicted latent class membership
save is cprob;
format is free;

## Appendix B

**Table A1:** Fit criteria for each competing model specification.

Models	Number of Day Level Classes				
	1 Class	2 Classes	3 Classes	4 Classes	5 Classes
**Fixed Effects Model**					
No. of free parameters	14	29	44	59	74
Log-likelihood	−173,793.306	−172,669.771	−172,039.204	−171,633.941	−171,377.292
AIC	347,614.612	345,397.542	344,166.407	343,385.883	342,902.585
BIC	347,728.092	345,632.608	344,523.06	343,864.121	343,502.409
aBIC	347,683.601	345,540.447	344,383.229	343,676.621	343,267.239
Lo-Mendell-Rubun LRT	--	<0.0001	<0.0001	<0.0001	<0.0001
Entropy	1	0.31	0.392	0.51	0.481
**Random Effects Model**					
2 individual level classes					
No. of free parameters		59	89	119	
Log-likelihood		−169,331.132	−168,700.96	−168,366.193	
BIC		339,258.502	338,301.338	337,934.968	
Entropy		0.581	0.569	0.555	
3 individual level classes					
No. of free parameters		89	134	179	
Log-likelihood		−166,936.279	−166,348.815	−166,062.761	
BIC		334,771.968	334,051.799	333,934.448	
Entropy		0.677	0.63	0.644	
4 individual level classes					
No. of free parameters		119	179		
Log-likelihood		−165,441.731	−164,845.696		
BIC		332,086.045	331,500.318		
Entropy		0.729	0.659		

Abbreviations: No, number; AIC, Akaike information criterion; BIC, Bayesian information criterion; Entropy, a pseudo-r-squared index; Lo-Mendel-Rubin LRT, likelihood ratio test comparing *q* classes models with *q-1* classes models. --: means no test.

Key model fitting parameters of the estimated latent classes (LC) models are shown in Appendix B Table A1. In the fixed effect LC models, the BIC declines as the number of classes increases. Using the MLCA models, the BIC improves largely. After examining the MLCA model results in greater detail, the model with three classes at the day level and three classes at the individual level was chosen as it enabled classification in interpretable, distinct classes. The solution that was suggested as optimal by the indices (2 classes at the day level shown in Appendix B Figure A1.; 4 classes at the individual level shown in Appendix B Figure A2.) was unable to separate high percentage CH and low percentage CH days and produced two individual level classes with nearly identical composition of days classes.
