# Supplementary material for: Day-Time Patterns of Carbohydrate Intake in Adults by Non-Parametric Multi-Level Latent Class Analysis—Results from the UK National Diet and Nutrition Survey (2008/09–2015/16)
Source: Nutrients. 2019 Oct 15;11(10):2476. doi: 10.3390/nu11102476 (PMC6835827; doi:10.3390/nu11102476)
Supplement: Supplementary file 1 [file nutrients-11-02476-s001.pdf]

Supplementary Tables for article: Day-time patterns of carbohydrate intake in adults by non-parametric Multi-level Latent Class Analysis – results from the UK National Diet and Nutrition Survey (2008/09 - 2015/16)

Table S1. Day level latent class solution (probabilities) for three classes LCA model. (No individual level model, n = 24483 observation days)

Time slots of the day	Responses of CH intake	n	%	HCHD (39.5%)	LCHD (20.4%)	RMD (40.1%)
6 am – 9 am	No energy intake	7655	31.2	0.129	0.450	0.320
	CH < 50%	4500	18.4	0.130	0.267	0.128
	CH ≥ 50%	12328	50.4	0.741	0.283	0.552
9 am – 12 noon	No energy intake	5447	22.2	0.237	0.079	0.401
	CH < 50%	7227	29.5	0.158	0.492	0.173
	CH ≥ 50%	11809	48.2	0.605	0.429	0.426
12 noon – 2 pm	No energy intake	4783	19.5	0.156	0.356	0.019
	CH < 50%	11112	45.4	0.405	0.413	0.56
	CH ≥ 50%	8588	35.1	0.439	0.231	0.421
2 pm – 5 pm	No energy intake	6926	28.3	0.13	0.123	0.659
	CH < 50%	8277	33.8	0.249	0.602	0.076
	CH ≥ 50%	9280	37.9	0.621	0.276	0.266
5 pm – 8 pm	No energy intake	3043	12.4	0.114	0.199	0.034
	CH < 50%	14240	58.2	0.516	0.59	0.639
	CH ≥ 50%	7200	29.4	0.37	0.211	0.328
8 pm – 10 pm	No energy intake	8722	35.6	0.322	0.291	0.48
	CH < 50%	8898	36.3	0.266	0.551	0.212
	CH ≥ 50%	6863	28.0	0.412	0.158	0.308
10 pm – 6 am	No energy intake	16295	66.6	0.68	0.59	0.751
	CH < 50%	4144	16.9	0.074	0.294	0.101
	CH ≥ 50%	4044	16.5	0.246	0.115	0.148

Abbreviations: LCA, latent class analysis; CH, carbohydrates; HCHD, high percentage carbohydrate day; LCHD, low percentage carbohydrate day; RMD, regular meals day. CH < 50% indicates that within the time slot, carbohydrate contributed < 50% total energy intake; CH ≥ 50% indicates that within the time slot, carbohydrate contributed ≥ 50% total energy intake.

Table S2. Means (standard deviations, sd), and counts (%) of the characteristics by different types of carbohydrate eating days (n = 24483).

	HCHD	LCHD	RMD	*P*-value[1]
n (%)	9667 (39.5)	5002 (20.4)	9814 (40.1)	

Country (%)				< 0.001
England	5627 (58.2)	2972 (59.4)	5291 (53.9)	
North Ireland	1194 (12.4)	527 (10.5)	1400 (14.3)	
Scotland	1527 (15.8)	813 (16.3)	1774 (18.1)	
Wales	1318 (13.6)	690 (13.8)	1349 (13.7)	
Day of Week (%)				< 0.001
Monday	1303 (13.5)	715 (14.3)	1370 (14.0)	
Tuesday	1266 (13.1)	674 (13.5)	1290 (13.1)	
Wednesday	1225 (12.7)	740 (14.8)	1233 (12.6)	
Thursday	1272 (13.2)	752 (15.0)	1425 (14.5)	
Friday	1458 (15.1)	797 (15.9)	1479 (15.1)	
Saturday	1537 (15.9)	703 (14.1)	1495 (15.2)	
Sunday	1605 (16.6)	621 (12.4)	1522 (15.5)	
Weekend, Yes (%)	3142 (32.5)	1324 (26.5)	3017 (30.7)	< 0.001
Total energy (kJ)	7539.9 (2875.9)	7160.2 (2922.2)	7439.7 (2978.9)	< 0.001
Carbohydrate (g)	222.8 (89.8)	209.7 (86.2)	206.6 (84.4)	< 0.001
Protein (g)	71.4 (29.8)	69.6 (30.2)	73.3 (32.9)	< 0.001
Fat (g)	65.4 (33.3)	63.94 (33.8)	67.24 (34.7)	< 0.001
Alcohol (g)	11.8 (27.3)	8.9 (24.3)	13.8 (33.0)	< 0.001
Total sugars (g)	98.6 (56.0)	88.0 (50.5)	86.4 (50.9)	< 0.001
Starch (g)	124.1 (55.8)	121.59 (56.1)	120.1 (54.6)	< 0.001
Non-Milk Extrinsic Sugar (g)	59.5 (49.3)	50.1 (43.4)	50.4 (44.8)	< 0.001
Fruit (g)	107.4 (137.9)	103.2 (129.1)	92.8 (126.0)	< 0.001
Yellow Red Green Vegetables (g)	26.5 (46.4)	26.8 (47.9)	26.2 (45.9)	0.681

[1] *P* values were obtained from χ^2 test for categorical variables, and one-way ANOVA comparing the means in multiple groups for continuous variables; CH, carbohydrate.

Abbreviations: HCHD, high percentage carbohydrate day; LCHD, low percentage carbohydrate day; RMD, regular meals day; NME sugar, non-milk extrinsic sugar. Non-milk extrinsic sugar is defined as: additional added free sugar, such as table sugar, honey, glucose, fructose and glucose syrups, sugars added to food and sugars in fruit juices.
